# Immune senescence in aged APP/PS1 mice

**DOI:** 10.1515/nipt-2023-0015

**Published:** 2023-08-14

**Authors:** Mai M. Abdelmoaty, Pravin Yeapuri, Jatin Machhi, Yaman Lu, Krista L. Namminga, Rana Kadry, Eugene Lu, Shaurav Bhattarai, Rodney Lee Mosley, Howard E. Gendelman

**Affiliations:** Department of Pharmacology and Experimental Neuroscience, College of Medicine, University of Nebraska Medical Center, Omaha, NE, USA; Department of Cellular and Integrative Physiology, University of Nebraska Medical Center, Omaha, NE, USA

**Keywords:** aging, Alzheimer’s disease, neuroinflammation, oxidative stress, Treg

## Abstract

**Objectives:**

To evaluate the linkage between age and deficits in innate and adaptive immunity which heralds both Alzheimer’s disease (AD) onset and progression. The pathobiological events which underlie and tie these outcomes remain not fully understood.

**Methods:**

To investigate age-dependent immunity in AD, we evaluated innate and adaptive immunity in coordinate studies of regulatory T cell (Treg) function, T cell frequencies, and microglial integrity. These were assessed in blood, peripheral lymphoid tissues, and the hippocampus of transgenic (Tg) amyloid precursor protein/presenilin 1 (APP/PS1) against non-Tg mice. Additionally, immune arrays of hippocampal tissue were performed at 4, 6, 12, and 20 months of age.

**Results:**

APP/PS1 mice showed progressive impairment of Treg immunosuppressive function with age. There was partial restoration of Treg function in 20-month-old mice. Ingenuity pathway analyses of hippocampal tissues were enriched in inflammatory, oxidative, and cellular activation pathways that paralleled advancing age and AD-pathobiology. Operative genes in those pathways included, but were not limited to triggering receptor on myeloid cells 1 (TREM1), T helper type 1 (Th1), and nuclear factor kappa-light-chain-enhancer of activated B cells (NF-κB) signaling pathways. Interleukin-17 (IL-17), nitric oxide, acute phase, and T cell receptor signaling pathways were also perturbed. Significant inflammation was observed at 6- and 12-months. However, at 20-months, age associated partial restoration of Treg function reduced inflammatory phenotype.

**Conclusions:**

Impaired Treg function, inflammation and oxidative stress were associated with AD pathology. Age associated partial restoration of Treg function in old mice reduced the hippocampal inflammatory phenotype. Restoring Treg suppressive function can be a therapeutic modality for AD.

## Introduction

The formation of amyloid plaques is a key pathologic feature of Alzheimer’s disease (AD). The amyloid precursor protein/presenilin 1 (APP/PS1) double transgenic (Tg) mouse model overproduces amyloid beta (Aβ), and as such, was used to interrogate AD pathology and for associated drug development research [1, 2]. Indeed, APP and PS1 harbor the mutations found in familial AD [3]. The AD models demonstrate age-related hippocampal dependent memory loss [4–7] and have uncovered relationships between progressive amyloid pathogenesis and hippocampal dysfunctions. However, the missing link between human AD and the APP/PS1 model are relationship(s) between peripheral immunity and brain immunity. These are each prominent features characteristic of human neurodegenerative disease [8]. While microglial neuroinflammation is a major contributor to age-linked AD pathogenesis [9, 10], the adaptive immunity also plays a prominent role in generating or exacerbating inflammatory responses in the AD aging brain [11, 12] and in other neurodegenerative diseases [13–15]. Specifically, microglia-CD4+ and -CD8+ T cell interactions are found in neurogenic niches where they generate inflammatory responses, affect neuronal vitality, and regulate neurogenesis [11]. Enhanced leukocyte recruitment that drives inflammatory responses remains operative in AD and a host of other neurodegenerative disorders that include Parkinson’s disease (PD), traumatic brain injury, and ischemic cerebrovascular pathologies [16]. Recently, we and others demonstrated parallel pathological insults in human and neurodegenerative disease models that involve effector T cells (Teffs), which facilitate brain injury-mediated neuroinflammation, microglia activation, increased amyloid burden, and neuronal loss leading to accelerated memory impairment [17–19]. Additionally, numbers of neurotoxic Teffs and amyloid plaque burden increase with age [19–21]. In contrast, CD4+ regulatory T cells (Tregs) are another T cell lineage that play a key role in immune tolerance and neuroprotection, and are also affected by age [22]. Specifically in AD and PD, misfolded aggregated self-proteins are able to break immune tolerance leading to the generation of autoreactive Teffs [23, 24]. Progressive, age-dependent accumulation of Teffs is associated with decreases in anti-inflammatory neuroprotective Tregs [23]. This overpowers Treg-mediated brain homeostasis and mitigation and leads to the progression of neurodegenerative disease [25–27].

The hippocampus represents a primary focal area of the brain affected by AD. The cellular and molecular profiles of the hippocampus during aging and progression of AD are not well-delineated. Moreover, the effects of Treg-mediated intervention on those hippocampal profiles are even less well-understood. In this study, we isolated hippocampi from brains of APP/PS1 Tg mice at 4-, 6-, 12-, and 20-months of age and compared those with age-matched non-Tg controls. We performed transcriptomic analyses focusing on innate and adaptive immune signatures. To evaluate the role of Tregs in AD pathology, we compared Treg number and function in blood, spleen, and lymph nodes of those mice. Based on each of these tests, Treg dysfunction, inflammatory, and oxidative stress profiles were uncovered throughout progressive AD in a relevant human disease model.

## Materials and methods

### Animals

Animal studies were approved by the Institutional Animal Care and Use Committee (IACUC) of the University of Nebraska Medical Center (UNMC). Transgenic mice overexpressing human APP695 with the Swedish mutation (Tg2576) were provided by Drs. Hsiao-Ashe and Carlson through the Mayo Medical Venture [28]. PS1 mice overexpressing human PS1 with M146L mutation were obtained from Dr. Duff through the University of South Florida [29]. Both strains were maintained on the B6;129 hybrid background. Male Tg2576 mice were crossbred with female PS1 mice to generate APP/PS1 double-Tg mice and non-Tg controls and produced in parallel [30, 31]. Female APP/PS1 mice and age-matched control mice were randomly divided into each potential age group at four months of age for biomarker analyses and immune cell-based comparisons.

### Flow cytometry analysis

At 4-, 6-, 12-, and 20-months of age, mice were terminally anesthetized with pentobarbital, blood collected by cardiac puncture in K_3_EDTA tubes (Greiner BioOne North America, 450475), and spleens removed and maintained in RPMI-1640 medium supplemented with 10 % fetal bovine serum and 1 % penicillin-streptomycin (10,000 U/mL). Mice were pericardially perfused with phosphate buffered saline (PBS), and axial, brachial, cervical, and inguinal lymph nodes and brains were harvested. Single cell suspensions of spleens and lymph nodes were prepared by maceration through a 70 μm mesh strainer as described [32]. Either 50 μL of blood or 1 × 10^6^ spleen or lymph node cells were stained for cell surface markers and assessed by flow cytometric analysis. Blood or immune cells were first reacted with fluorescently conjugated antibodies to CD3e-PE (eBioscience, 12003181), CD8a- PE-Cyanine 5.5 (eBioscience, 35008180), CD4-APC-H7 (BD Pharmingen, 560181), and CD25-PE-Cy7 (eBioscience, 25025182) in PBS/1 % FCS/0.9 % NaN_3_ at 4 °C for 30 min. Cells were fixed and permeabilized using transcription factor staining buffer kit (eBioscience, 552300) for 45 min at 4 °C then incubated with anti-FOXP3-Alexa Fluor 488 (BioLegend, 320012). Fluorescence-minus-one controls for each antibody were used during flow cytometric analysis to ensure accurate gating of cell populations.

### Treg functional assays

CD4+CD25+ Tregs and CD4+CD25− T responder cells (Tresps) were isolated from mice spleens using EasySep mouse Treg enrichment kit (Stemcell Technologies, 18783) following the manufacturer’s instructions and as described [32]. Briefly, from single cell suspensions, CD4+ T cells were enriched by negative selection using EasySep (Stemcell) mouse CD4+ T cell isolation cocktail from which CD25+ cells were then positively selected using EasySep (Stemcell) mouse CD25+ Treg selection cocktail. Isolated CD4+CD25+ cells were more than 90 % FOXP3+ by flow cytometric analysis. The CD4+CD25− Tresp isolates from non-Tg control spleens were more than 90 % pure and were labeled with carboxyfluorescein succinimidyl ester (CFSE) (Thermo Fisher Scientific, C34554) for use in proliferation assays for Treg function. CD4+CD25+ Tregs from different experimental groups were serially diluted by 2-fold in a U-bottom 96-well plate to obtain 50, 25, 12.5, and 6.25 × 10^3^ Tregs in 100 μL of media/well. Added to each well were 50 × 10^3^ CFSE-labeled Tresps to obtain Tresp:Treg ratios of 1:1, 1:0.5, 1:0.25 and 1:0.125, respectively, while wells only with Tresps served as positive proliferation controls. Mouse T cell activating CD3/CD28 Dynabeads (Thermo Fisher Scientific, 11456D) were added to each well at Tresp:bead ratio of 1:1 to induce proliferation of Tresps. After 72 h incubation at 37 °C, the Treg-mediated immunosuppressive function seen as the ability to inhibit the proliferation of CFSE-stained Tresps and loss of CSFE dye, was determined using flow cytometric analysis and reported as Treg-mediated percent inhibition.

### Immunohistochemistry

After terminal anesthesia and transcardial perfusion with PBS, brains were immediately harvested and divided into two hemispheres. The left hemisphere was immediately frozen on dry ice for biochemical analysis and the right was processed for immunohistochemistry [19]. Briefly, the right hemisphere was immersed in 4 % paraformaldehyde/PBS for 48 h at 4 °C and cryoprotected by immersion in 15 % then 30 % sucrose for 24 h/immersion at 4 °C. Fixed brain sections were embedded and coronally sectioned with a cryostat (Thermo Fisher Scientific) at 30 μm sections and stored at −80 °C. Immunohistochemistry was performed using antibodies against ionized calcium-binding adapter molecule 1 (Iba1) (1:1,000, rabbit polyclonal, Wako Chemicals, 01919741) and pan-Aβ (1:500, rabbit polyclonal, Thermo Fisher Scientific, 715800). Biotin-conjugated anti-rabbit secondary antibody was used for immunodetection followed by a tertiary incubation with Vectastain ABC Elite kit (Vector Laboratories, PK6100). One percent Thioflavin-S in 50 % ethanol was used for counterstaining compact amyloid plaques (Sigma-Aldrich, T1892). For each animal, six sections/slide were collected and stained. Slides were masked and coded, and Aβ occupied area was determined using the Cavalieri Estimator probe (grid spacing 15 μm), while the number of Iba1-reactive microglia were counted using the Optical Fractionator probe of Stereo Investigator (MBF Bioscience) as described [31]. Briefly, Aβ and microglia were quantified with a high-sensitivity digital camera (OrcaFlash2.8, Hamamatsu C11440-10C, Hamamatsu, Japan) interfaced with a Nikon Eclipse 90i microscope (Nikon, Melville, NY, USA). For quantitation of each section, the region of interest was traced by electronic bit map and a counting frame was applied. The dimensions for the counting frame were 120 × 100 μm, and 245 × 240 μm for the grid size. The z-plane focus was adjusted at each section for clarity. Reactive microglia (Iba-1+ and amoeboid) were quantified by the fractionator with marked positive cells in each counting frame. Based on the set parameters and marked cell counts, Stereo Investigator computed cell population estimates for each animal.

### Transcriptomic and pathway enrichment assays

Hippocampal tissues of left hemisphere were collected from mice of different groups. Total RNA was isolated using RNeasy Mini Kit (Qiagen, catalog no. 74104), and cDNA was generated utilizing RevertAid First Strand cDNA synthesis kit (Thermo Fisher Scientific, catalog no. K1622) followed by amplification and quantification using RT^2^ Profiler Mouse Innate and Adaptive Immune Response 96-well Array (Qiagen, catalog no. 330231) with RT^2^ SYBR Green ROX qPCR Mastermix (Qiagen, catalog no. 330523). The qPCR cycling conditions were 95 °C for 10 min for 1 cycle, followed by 40 cycles of 95 °C for 15 s and 60 °C for 1 min using Eppendorf Mastercycler ep realplex 2S. Fold changes were determined with Qiagen’s RT^2^ Profiler analysis software. Gene ontology (GO) annotation of screened genes was conducted using the Cytoscape ClueGO to identify the enriched immune responses, biological processes, cellular components, molecular functions, and Reactome reactions and pathways [33]. Ingenuity Pathway Analysis (IPA) (Qiagen) was used to identify and compare the pathways enriched in two different comparison analyses: (1) For APP/PS1 and control mice, age-variable comparisons at 6, 12, and 20 months of age were determined; and (2) for interactions of age and AD pathology, APP/PS1 mice at 6, 12, and 20 months of age were compared to 4 months old group. IPA was used to identify the pathways enriched in 4, 6, 12, and 20 months of age in the APP/PS1 and control mice. Functional and pathway enrichment analyses of upregulated and downregulated genes in age and AD pathology-variable groups were conducted using Cytoscape ClueGO for GO terms, Kyoto Encyclopedia of Genes and Genomes (KEGG), and Reactome. The Search Tool for the Retrieval of Interacting Genes/Proteins (STRING) local network cluster enrichment analysis was conducted using Cytoscape string App (https://apps.cytoscape.org/apps/stringapp), which provides functional protein-protein interactions (PPIs), including direct (physical) and indirect (functional) associations [34].

### Statistical analysis

Results are presented as the mean ± standard error of the mean (SEM). Student’s t-test or one-way analysis of variance (ANOVA) was used to analyze differences in the mean values between groups. P values ≤0.05 were considered statistically significant. Statistical analysis was performed using GraphPad Prism 9.4.1 software (GraphPad Software, San Diego, CA). IPA was performed using analysis criteria: mouse as species, hippocampus and central nervous system (CNS) cell lines as tissues, and absolute fold changes ≥1.25. IPA comparison analyses were performed using cellular and humoral immune responses, cellular stress and injury, cytokine signaling, ingenuity toxicity lists, neurotransmitters and other nervous system signaling, and transcriptional regulation for selecting the enriched signaling pathways in different comparisons. Selection criteria for the enriched pathways were −log_10_ (p value) ≤1.3 (i.e., p value ≤0.05) and absolute z-score ≥2. ClueGO analysis was performed using p value ≤0.05 to select the enriched pathways.

## Results

### APP/PS1 mice develop age-associated increase in amyloid deposition and microglia activation

**Figure 1:**
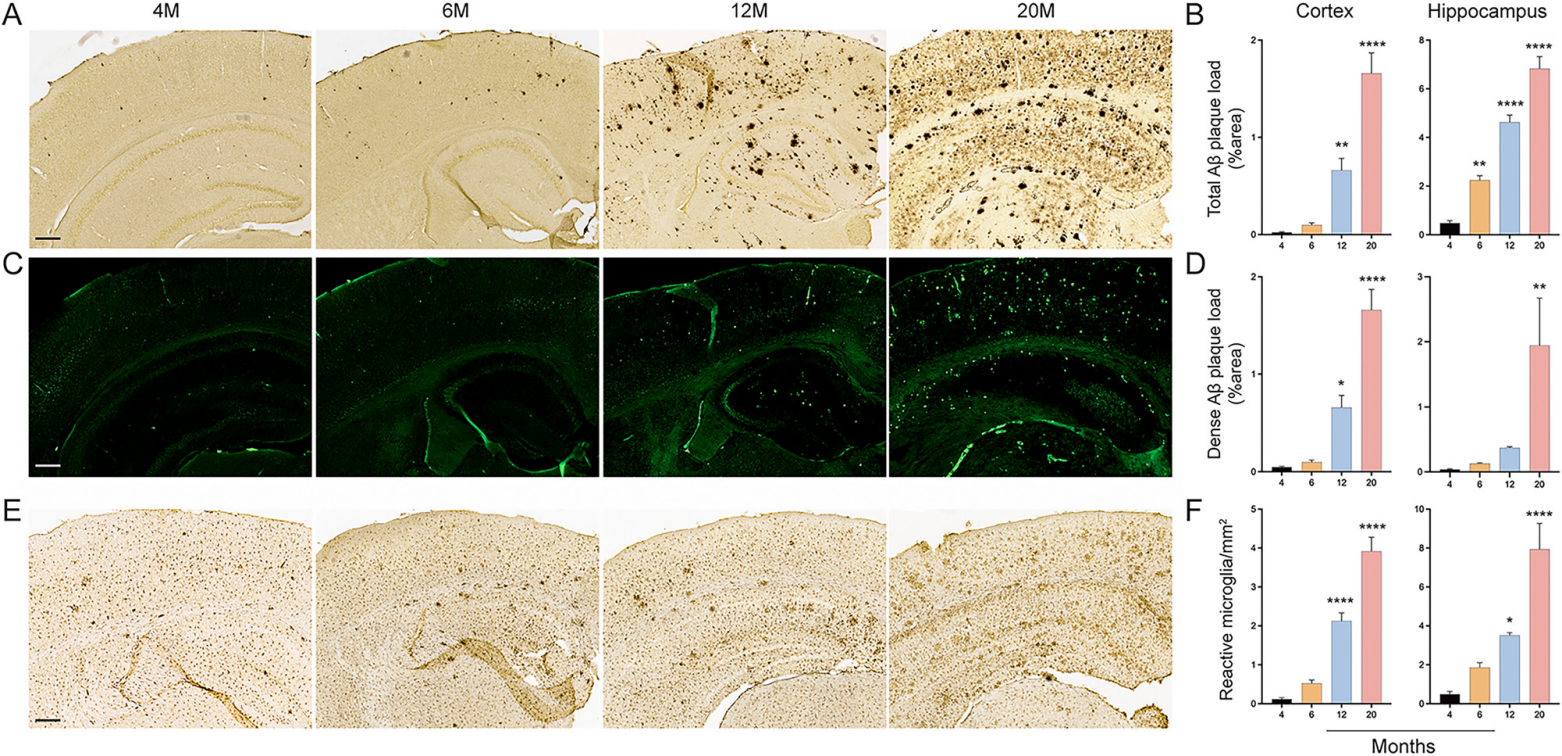
Amyloid deposition and microglia activation throughout AD progression. Immunohistochemistry (pan-Aβ) and immunofluorescence (Thioflavin-S) were performed in APP/PS1 mice at 4-, 6-, 12-, and 20-months of age to determine the area occupied by Aβ plaques in cortical and hippocampal brain sections. (A) Representative images showing total amyloid plaques in sections of cortical and hippocampal brain regions (scale bar=100 μm). (B) Percentage of area within cortices and hippocampi occupied by total Aβ plaques. (C) Representative images showing dense amyloid plaques in sections of cortical and hippocampal brain regions (scale bar=100 μm). (D) Percentage of area within cortices and hippocampi occupied by dense Aβ plaques. Numbers of reactive Iba1+ microglia cells in cortical and hippocampal brain regions were quantified by immunohistochemistry. (E) Representative images showing Iba1+-reactive cells in cortical and hippocampal (scale bar=100 μm). (F) Number of reactive microglia as Iba1+ ameboid cells in cortical and hippocampal tissues. Statistical differences between groups were determined using one-way ANOVA followed by Dunnett’s multiple comparisons test for n=4 mice/group whereby ^*^p≤0.05, ^**^p≤0.001, ^***^p≤0.0001, and ^****^p≤0.00001.

APP/PS1 mice develop amyloid plaque in the brain as early as three months of age [35], thus we evaluated the amyloid plaque deposition in the APP/PS1 mice brains by immunohistochemistry and immunofluorescence which showed remarkable increases in amyloid plaque deposition in the cortices and hippocampi of 12- and 20-month old mice (Figure 1A). APP/PS1 mice of 6, 12, and 20 months of age showed total Aβ (pan-Aβ) loads in the cortices (4-fold, p=0.948; 27-fold, p=0.008; and 67-fold, p≤0.0001; respectively) compared to 4-month mice (Figure 1B). In addition, 6-, 12-, and 20-month old mice showed total amyloid plaque loads in hippocampi (5-fold, p=0.0042; 10-fold, p≤0.0001; and 14-fold, p≤0.0001, respectively) compared to 4-month old mice. Similarly, 6-, 12-, and 20-month old APP/PS1 mice showed dense Aβ (Thioflavin-S) loads in cortical tissues (2-fold, p=0.9822; 14-fold, p=0.0106; and 34-fold, p≤0.0001, respectively) compared to 4-month old mice (Figure 1C). Additionally, 6-, 12-, and 20-month old mice showed dense amyloid plaque loads in hippocampal tissues of (3-fold, p=0.9961; 10-fold; p=0.8556; and 50-fold; p=0.0083, respectively) compared to 4-month old mice (Figure 1D). Thus, amyloid plaque depositions suggest that the age-associated amyloidosis in APP/PS1 mice increase throughout disease progression.

Microglia activation is a common hallmark signature of neuroinflammation in AD patients and animal models [36], therefore we assessed numbers of reactive microglia (Iba1-reactive cells with amoeboid morphology) in hippocampi and cortices of APP/PS1 mice at different ages. We found that numbers of Iba1+ amoeboid cells increase over time throughout disease progression in the cortex in 6-, 12-, and 20-month old mice (4-fold, p=0.4246; 18-fold, p≤0.0001; and 33-fold, p≤0.0001, respectively) compared to those at 4 months of age (Figure 1E and F). Furthermore, numbers of Iba1+ amoeboid cells increase with age throughout disease progression in hippocampi of 6-, 12-, and 20-month old mice (4-fold, p=0.3871; 7-fold, p=0.0221; and 16-fold, p≤0.0001, respectively) compared to 4-month old group (Figure 1E and F). These data demonstrate that age-associated microglial activation increases with progression of AD pathology in APP/PS1 mice.

### APP/PS1 mice show inflammation and diminution of Treg function throughout progressive AD

**Figure 2:**
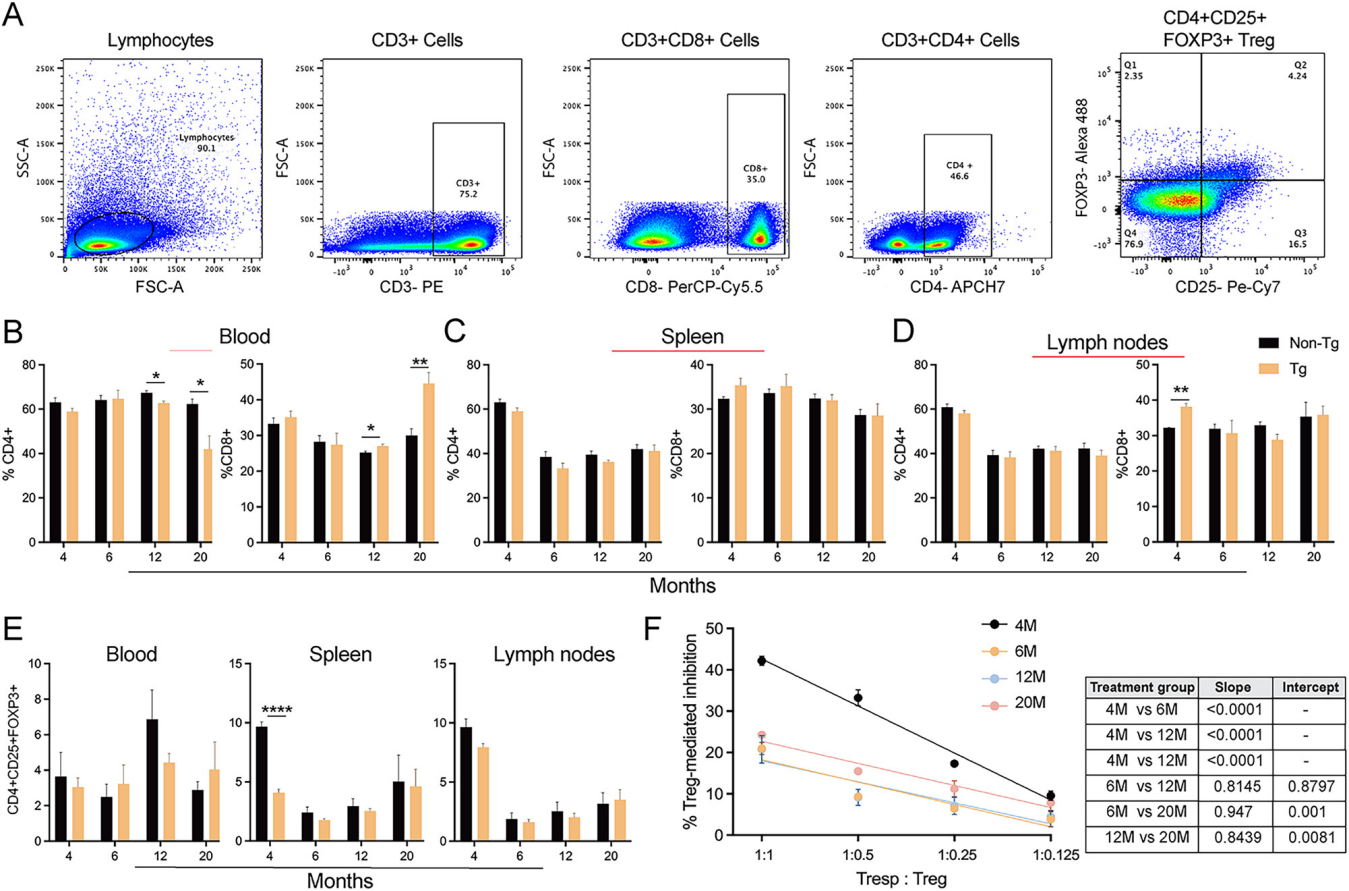
Treg frequency and function throughout progressive AD. (A) Flow cytometry gating strategies for T lymphocyte analysis from APP/PS1 and non-Tg controls. Frequencies of CD4+ and CD8+ T cells within total T lymphocyte population in blood (B), spleen (C), and lymph nodes (D) for 4-, 6-, 12-, and 20-month old APP/PS1 and non-Tg mice. (E) Frequencies of CD4+CD25+FOXP3+ Tregs within CD3+CD4+ T cells in blood, spleen, and lymph nodes of non-Tg and Tg mice at different ages. (F) Inhibition of CFSE-stained-Tresp (CD4+CD25-) proliferation by Tregs from APP/PS1 at different ages. Significant differences (table) in slope and intercept were determined by linear regression analysis. (B–F) Data are represented as mean ± SEM for n=3–4 mice per group. Statistical significance between groups was determined using Student’s t-test or one-way ANOVA followed by Tukey’s multiple comparison test. The p value ≤0.05 was considered significant; ^*^p≤0.05, ^**^p≤0.001, ^***^p≤0.0001, and ^****^p≤0.00001. M, months; non-Tg, control; Tg, APP/PS1.

Basic T cell phenotype profiles in peripheral blood, spleens, and lymph nodes were assessed by flow cytometric analysis for age-matched APP/PS1 Tg and non-Tg controls. Gating strategies for different T cell lineages were applied to obtain T cell lineage profiles (Figure 2A). Peripheral blood CD4+ lymphocyte frequencies were significantly decreased in 12- and 20-month groups compared to control mice (p=0.0124, p=0.0184, respectively), while CD8+ lymphocyte frequency significantly increased in 12- and 20-month APP/PS1 mice compared to non-Tg controls (p=0.0213, p=0.0069, respectively) (Figure 2B) suggesting an increased cytotoxic and pro-inflammatory environment throughout AD progression. Frequencies of CD4+ and CD8+ lymphocytes did not change in the splenic and lymph node tissues between APP/PS1 and control groups at 6, 12, and 20 months of age, but CD8+ T cells were significantly increased in lymph nodes of 4-month APP/PS1 Tg mice compared to controls (p=0.004), while frequencies of CD4+ lymphocytes diminished in spleen and lymph nodes of APP/PS1 and control mice (Figure 2C and D). Similarly, frequency of CD4+CD25+FOXP3+ Tregs significantly diminished in the spleens of 4-month-old APP/PS1 mice (p<0.0001), but did not significantly change in any other tissues at any ages compared to non-Tg controls (Figure 2E). Furthermore, splenic Tregs were isolated from APP/PS1 mice of different age groups, and their immunosuppressive function was compared by evaluating the suppressive effect on the proliferation of CFSE-labeled Tresp cells isolated from non-Tg mice. We evaluated Treg mediated inhibition of Tresp proliferation three days after co-culture with increasing dilution of Tregs [37, 38]. There was significant reduction in Treg-mediated immunosuppressive function in 6-, 12-, and 20-month-old APP/PS1 mice compared to 4-month-old APP/PS1 mice, however, we observed increased Treg function in 20-month-old mice compared to 6- and 12-month-old APP/PS1 mice (Figure 2F). These data indicate an overall increase in Treg dysfunction with age throughout AD progression with minor restoration of Treg function in aged mice.

### Functional and pathway enrichment analyses of hippocampal immune responses in APP/PS1 mice with progressive AD pathologies

To investigate alterations in immune responses and functions within the hippocampus as a function of age, a total of 84 key immune-related genes were examined. Transcriptional changes linked to immunity were screened using RT^2^ Profiler Mouse Innate and Adaptive Immune Response Array. GO annotation of screened genes showed 87.5 % and 72.9 % enrichment of positive regulation of humoral immune response (immune response and biological process analyses, respectively), and 84.6 % enrichment of spontaneous hydrolysis of C3 thioester (Reactome analysis) (Supplementary Figure 1). Different analysis terms for immune responses, biological processes, cellular components, molecular functions, and Reactome reactions and pathways are indicated in Data File 1. Together, this suggests the enrichment of various immune responses and complement cascade activities.

#### Non-Tg mice show age associated neurodevelopmental and anti-inflammatory immune phenotype in the hippocampus

**Figure 3:**
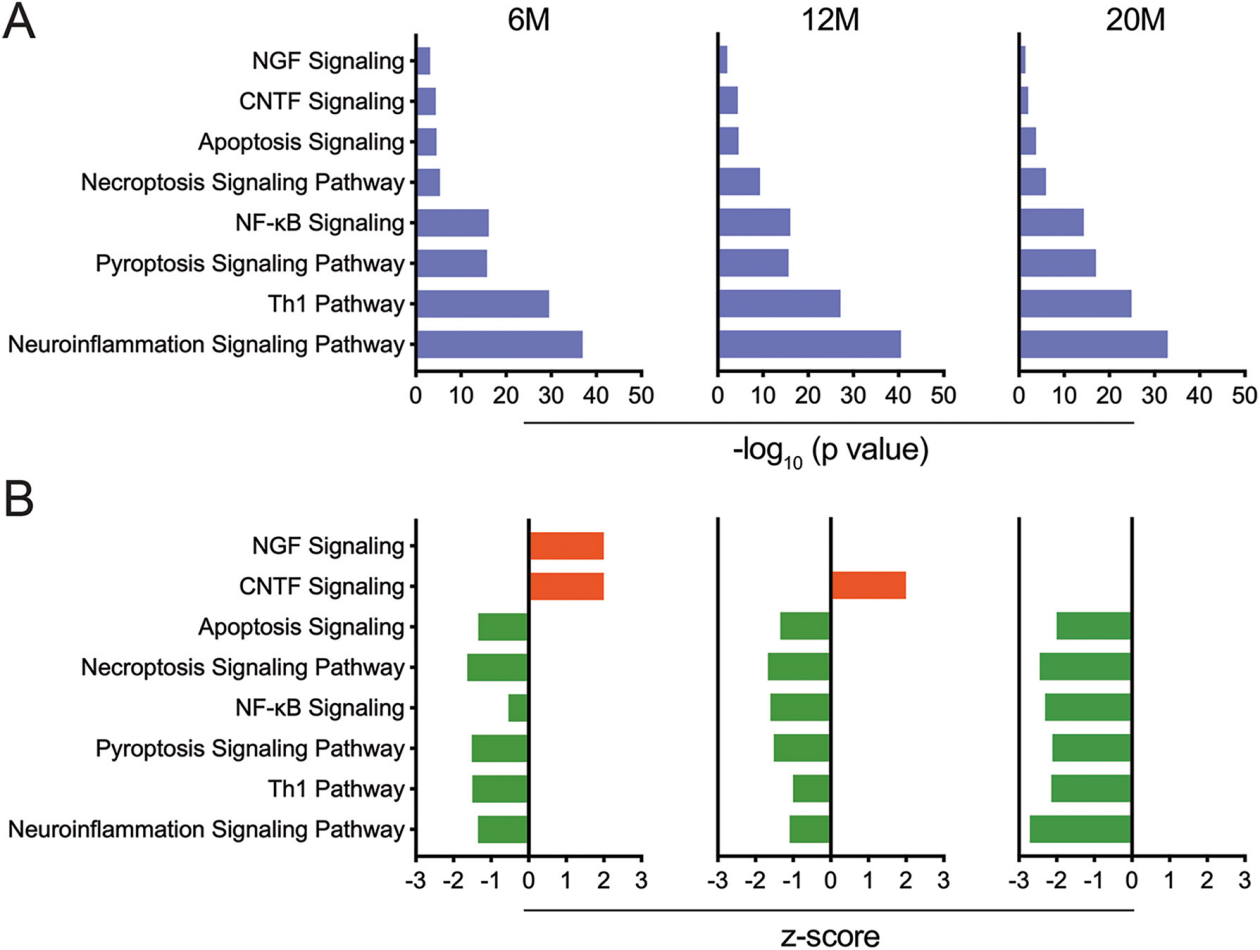
Canonical pathway enrichments of immune-related genes in hippocampi of non-Tg control mice. Hippocampal tissues were obtained from non-Tg control mice at 4-, 6-, 12- and 20-months of age (4M, 6M, 12M, and 20M). Canonical pathway enrichment analysis for hippocampal tissues was performed using IPA (Qiagen) for 6M, 12M, and 20M non-Tg control mice compared to 4M non-Tg controls. (A) Data was represented using −log_10_ (p value), where p<0.05 was considered significant. (B) Data represented using z-score where z-score ≤−2 or ≥2 were considered significant. Inhibited pathways are represented in green, whereas activated pathways are represented in red.

To evaluate age associated hippocampal immune changes in non-Tg mice, expression of adaptive and innate immune response genes in 6-, 12-, and 20-month non-Tg mice were compared against 4-month non-Tg mice. A normal or downregulated expression of several screened genes was observed with increasing age (Supplementary Figure 2 and Data File 2), and there was a marked absence of cellular activation in non-Tg mice with progression of age. IPA comparison analysis of canonical pathways altered at different ages, compared to 4-month old non-Tg mice, showed significant enrichment of neuronal development signaling pathways until 12-months of age including nerve growth factor (NGF) and ciliary neurotrophic factor (CNTF) signaling pathways, as well as inflammatory and cell death signaling pathways including neuroinflammation, T helper type 1 (Th1), nuclear factor kappa-light-chain-enhancer of activated B (NF-κB), apoptosis, necroptosis, and pyroptosis signaling pathways (Figure 3A and Data File 3). The NGF signaling pathway in 6-month non-Tg mice (z-score=2, p=6.56 × 10^−4^) and the CNTF signaling pathway in 6- and 12-month non-Tg mice (z-score=2, p=3.67 × 10^−5^; and z-score=2, p=3.98 × 10^−5^, respectively) were significantly changed (Figure 3B and Data File 3), indicating that normal functions of neurotrophic and nerve growth factors for the neuronal development are activated in young and middle age brains. Several pathways, such as inflammatory and cell death signaling pathways, were inhibited at all ages of non-Tg control mice (Figure 3B and Data File 3), suggesting that those pathways were homeostatic hippocampal activities.

**Figure 4:**
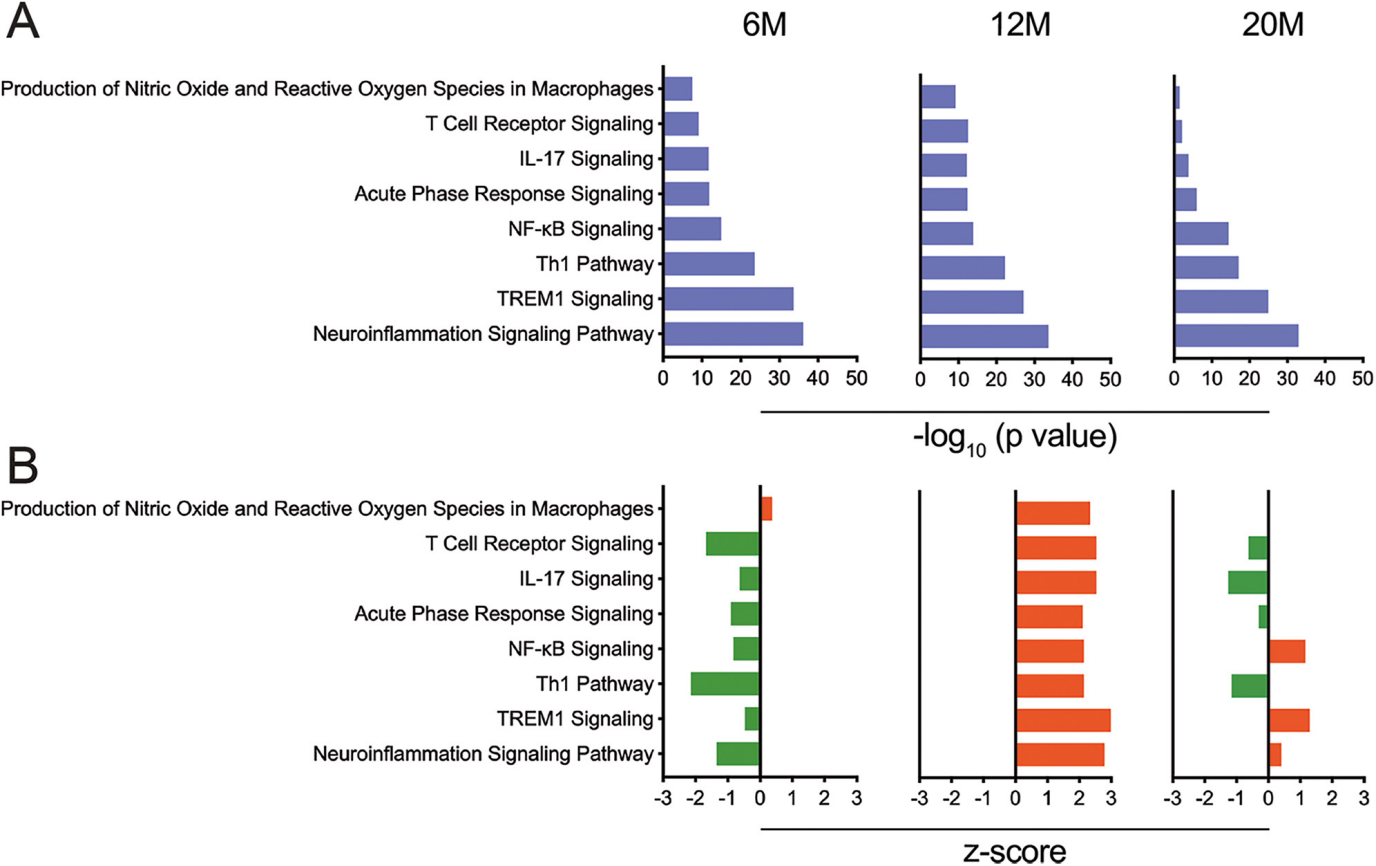
Canonical pathway enrichments of immune-related genes in hippocampi of APP/PS1 mice. Hippocampal tissues were obtained from APP/PS1 mice at 4-, 6-, 12- and 20-months of age (4M, 6M, 12M, and 20M). Canonical pathway enrichment analysis was performed using IPA (Qiagen) for 6M, 12M, and 20M APP/PS1 mice and compared to 4M APP/PS1 mice. (A) Data represented as −log_10_ (p value), whereby p values ≤0.05 was considered significant. (B) Data represented as z-score, whereby z-scores ≤−2 or ≥2 were considered significant. Inhibited pathways are represented in green, whereas activated pathways are represented in red.

#### APP/PS1 mice showed age associated increase in neuroinflammation and pro-inflammatory T cell responses in the hippocampus

To evaluate age associated hippocampal immune changes with progressive AD, expression of adaptive and innate immune response genes in 6-, 12-, and 20-month old APP/PS1 mice were compared against 4-month old APP/PS1. At 6-months of age, there was no significant differences in the gene profile compared to 4-month old APP/PS1 mice (Supplementary Figure 3). However, IPA analysis showed upregulation of nitric oxide and reactive oxygen species in macrophages. Additionally, many of the neuroinflammatory pathways such as Th1 pathway and neuroinflammatory signaling pathway were negatively enriched compared to 4-month APP/PS1 mice indicating early anti-inflammatory phenotype activated in response to increasing Aβ deposition (Figure 4, Supplementary Figure 3 and Data File 4). Table 1 shows the z and p values of enriched pathways associated with age-related disease progression in APP/PS1 mice compared to 4-month old APP/PS1 mice. In 12-month old mice, several screened genes were activated, suggesting significant changes in the immune phenotype that corelated with significantly increased Aβ deposition (Supplementary Figure 3 and Data File 4). IPA analysis showed significant upregulation of production of nitric oxide and reactive species. In addition, significant upregulation of neuroinflammatory pathways along with pro-inflammatory cellular immune responses such as T cell receptor, IL-17, NF-κB and Th1 signaling were detected (Figure 4B). Interestingly, in 20-month old mice, several upregulated genes appear to be downregulated in spite of increased Aβ deposition and reactive microglia in old mice (Supplementary Figure 3 and Data File 4). IPA analysis showed downregulation of cellular immune responses such as T cell receptor signaling, IL-17 and Th1 pathway while neuroinflammatory signaling, NF-κB and TREM-1 signaling remained upregulated, but with a lower Z-score compared to 12 month old APP/PS1 mice (Figure 4B, Supplementary Figure 3 and Data File 4). Together the data indicate that neuroinflammation and pro-inflammatory cellular immune responses increase with age with peak levels observed in 12-month old mice. However, at 20-months, mice show downregulation of pro inflammatory cellular immune responses compared to 12-month mice while still expressing a neuroinflammatory phenotype.

#### Genes involved in AD pathogenesis progression in APP/PS1 mice: comaprison with 4-month APP/PS1 mice

Differential gene expression analysis of the aforementioned pathways was performed from hippocampal tissues of AD mice at different ages to reveal potential genes linked to AD pathogenesis and progression. The mitogen-activated protein kinase (MAPK) signaling pathway is closely associated with the neuronal apoptosis in AD [39]. MAPK pathways induced by T cell receptor stimulation are associated with T cell immune responses [40, 41]. Here, expression of *MAPK1* gene was significantly upregulated at 6- and 12-months of age (3-fold, p=4.09 × 10^−4^ and 2-fold, p=0.026, respectively) (Data File 4). Additionally, toll-like receptors (TLRs) are pattern recognition receptors (PRRs) implicated in neuroinflammation and AD pathogenesis [42]. TREM-1 amplifies TLR-induced inflammation by increasing the production of pro-inflammatory cytokines [43]. Our data showed that genetic expression of *TLR2* increased at 6-, 12-, and 20-months of age (8-fold, p=0.048; 19-fold, p=0.140; and 8-fold, p=0.192, respectively) (Data File 4). Moreover, expression of the myeloid differentiation primary response 88 (*MYD88*) gene expression, an intermediary of TLR signaling, is increased in the blood of double transgenic APP/TAU mice used as AD animal model [43, 44]. TLR-induced IL-1 made naïve CD4+ T cells unresponsive to Treg-mediated suppression and led to Th1 cell differentiation [45]. In this study, *MYD88* gene expression in hippocampus increased, but not significantly, at 20-months of age after disease pathology was clearly observed (1.3-fold, p=0.44) (Data File 4). Moreover, NF-κB regulates survival, activation, and differentiation of innate and adaptive inflammatory cells [46]. NF-κB also binds to sites in the promoter regions of genes involved in amyloidogenesis and inflammation [47]. Here, we found expression of *NF-κB1* elevated at 12-months, but not significantly (2.8-fold, p=0.129) (Data File 4). Recently, CD8+ T cells were found localized to brains of APP/PS1 mice [21]. Twelve- and 20-month APP/PS1 mice showed increased CD8+ lymphocyte numbers in blood (Figure 2B) and were associated with cells in brains of APP/PS1 mice. The upregulation of *MAPK1*, *TLR2*, *MYD88*, *NF-κB1* in AD coordinated with T cell receptor and Th1 signaling activation seen in aged PP/PS1 mice, was associated with the development of neuroinflammatory responses.

Interestingly, functional and pathway enrichment analyses of upregulated and downregulated genes by Cytoscape ClueGo GO annotation at 12- and 20-months of age that coincided with AD pathology, found the pathway for the spontaneous hydrolysis of C3 thioester was 75 % enriched (Supplementary Figure 4). As the complement cascade is a critical component of the innate immune system and contributes to the inflammatory response, enrichment of spontaneous hydrolysis of C3 thioester in 12- and 20-month old APP/PS1 mice suggests the involvement of the complement cascade in inflammatory responses found at both ages. Thus, these data may underlie the importance of complement in AD and other neurodegenerative diseases [48]. The pathways for biological processes, cellular components, molecular functions, and Reactome reactions for each function are indicated at different ages in Data File 6. The PPIs related to five categories (biological processes, cellular components, molecular functions, KEGG pathways, and Reactome pathways) were also identified using STRING analysis of their interaction networks for transcriptomic data at different ages (Data File 7). Those PPIs demonstrate a complex immune-, inflammatory-, and oxidative stress-related interactive network that comprise and drive disease progression in AD.

#### AD progression at different ages in APP/PS1 mice: comparison with age matched non-Tg mice

To normalize for age and isolate immune phenotype associated with AD progression, APP/PS1 mice from each age group were compared against age matched non-Tg mice. The gene expression profile showed significant changes in immune phenotype with age (Data File 8 and Supplementary Figure 5). At 4-months of age, multiple pro-inflammatory cytokines and chemokines were downregulated such as C-C motif chemokine receptor 5 (*CCR5*; −9-fold, p=0.35), C-reactive protein (*CRP*; −5-fold, p=0.736), colony stimulating factor 2 (*CSF2*; −3-fold, p=0.902), C-X-C motif chemokine ligand 10 (*CXCL10*; −5-fold, p=0.62), *CXCL8* (−3-fold, p=0.373), *IL1B* (−2-fold, p=0.593), *IL23A* (−4-fold, p=0.743), nucleotide-binding oligomerization domain-containing protein 2 (*NOD2*; −3-fold, p=0.666), and tumor necrosis factor-α (*TNF*; −5-fold, p=0.643). Thus, trending absence of cellular activation in 4-month old APP/PS1 mice suggests that disease process is not yet initiated or is cryptic. At 6-months of age, several pro-inflammatory cytokines and chemokines were trending to be upregulated by at least 2-fold including *CCR4* (2-fold, p=0.256), *CXCL10* (3-fold, p=0.225), *CXCR3* (6-fold, p=0.053), *CRP* (3-fold, p=0.279), and *TLR2* (2.5-fold, p=0.169), suggesting the initiation of cellular activation and inflammation in 6 month old APP/PS1 mice. While by 12 months of age, multiple pro-inflammatory cytokines and chemokines as well as TLRs were trending to be upregulated by greater than 2-fold, and included *CCL2* (6-fold, p=0.11), *CCR4* (3-fold, p=0.277), *CCR5* (5-fold, p=0.259), *CRP* (4-fold, p=0.135), *CXCL10* (3-fold, p=0.276), *CXCR3* (10-fold, p=0.199), *CXCL8* (4-fold, p=0.261), *IL23A* (3-fold, p=0.273), NOD-like receptor (NLR) family pyrin domain containing 3 (*NLRP3*; 3-fold, p=0.022), *NOD2* (2.5-fold, p=9.57 × 10^−3^), *TNF* (2.3-fold, p=0.296), *TLR7* (4-fold, p=0.261), and *TLR8* (5-fold, p=0.259). This advanced the notion that cellular activation and inflammation were more exacerbated in APP/PS1 mice after 12-months of age. Similarly, at 20-months of age, more genes for pro-inflammatory cytokines, chemokines, and TLRs trended to greater than 2-fold upregulation expression for *CCR4* (4.5-fold, p=0.321), *CCR5* (10-fold, p=0.278), *CXCL8* (3-fold, p=0.362), *CRP* (12-fold, p=0.361), *IL17A* (3-fold, p=0.38), *IL18* (5-fold, p=0.364), *IL23A* (6-fold, p=0.273), *NLRP3* (3-fold, p=0.236), *NOD2* (2.4-fold, p=0.383), *TNF* (6-fold, p=0.167), *TLR2* (7-fold, p=0.33), *TLR4* (3-fold, p=0.135), *TLR6* (3-fold, p=0.35), and *TLR8* (8-fold, p=0.23). This trend of increased cellular activation and inflammation by 20-months of age suggested an advanced spread of disease exacerbation in those APP/PS1 mice.

**Table 1:** Inflammation and oxidative stress-mediated signaling pathways in APP/PS1 mice of different ages.

Canonical pathways	6-month old		12-month old		20-month old	
	z-score	p-Value	z-score	p-Value	z-score	p-Value
Th1 pathway	−2.138	2.52 × 10^−24^	2.138	1.41 × 10^−25^	−1.155	6.46 × 10^−23^
T cell receptor signaling	−1.667	8.45 × 10^−10^	2.53	3.38 × 10^−12^	−0.632	3.10 × 10^−13^
TREM1 signaling	−0.471	2.20 × 10^−34^	2.982	8.14 × 10^−36^	1.291	7.06 × 10^−28^
Neuroinflammation signaling	−1.347	7.69 × 10^−37^	2.785	7.19 × 10^−41^	0.408	2.01 × 10^−34^
IL-17 signaling	−0.632	2.03 × 10^−12^	2.53	6.31 × 10^−12^	−1.265	7.32 × 10^−13^
NF-κB signaling	−0.832	1.09 × 10^−15^	2.138	1.35 × 10^−16^	1.155	1.20 × 10^−14^
Acute phase response signaling	−0.905	1.23 × 10^−12^	2.111	4.33 × 10^−12^	−0.302	3.98 × 10^−13^
Production of NO and ROS in macrophages	0.378	3.24 × 10^−8^	2.333	1.68 × 10^−10^	0	5.86 × 10^−10^

All comparisons were evaluated against 4-month old APP/PS1 mice. The absolute z-score ≥2 and/or p value ≤0.05 are considered significant. A positive z-score is upregulation, a negative z-score is downregulation, z-score of zero is no change in activity.

**Figure 5:**
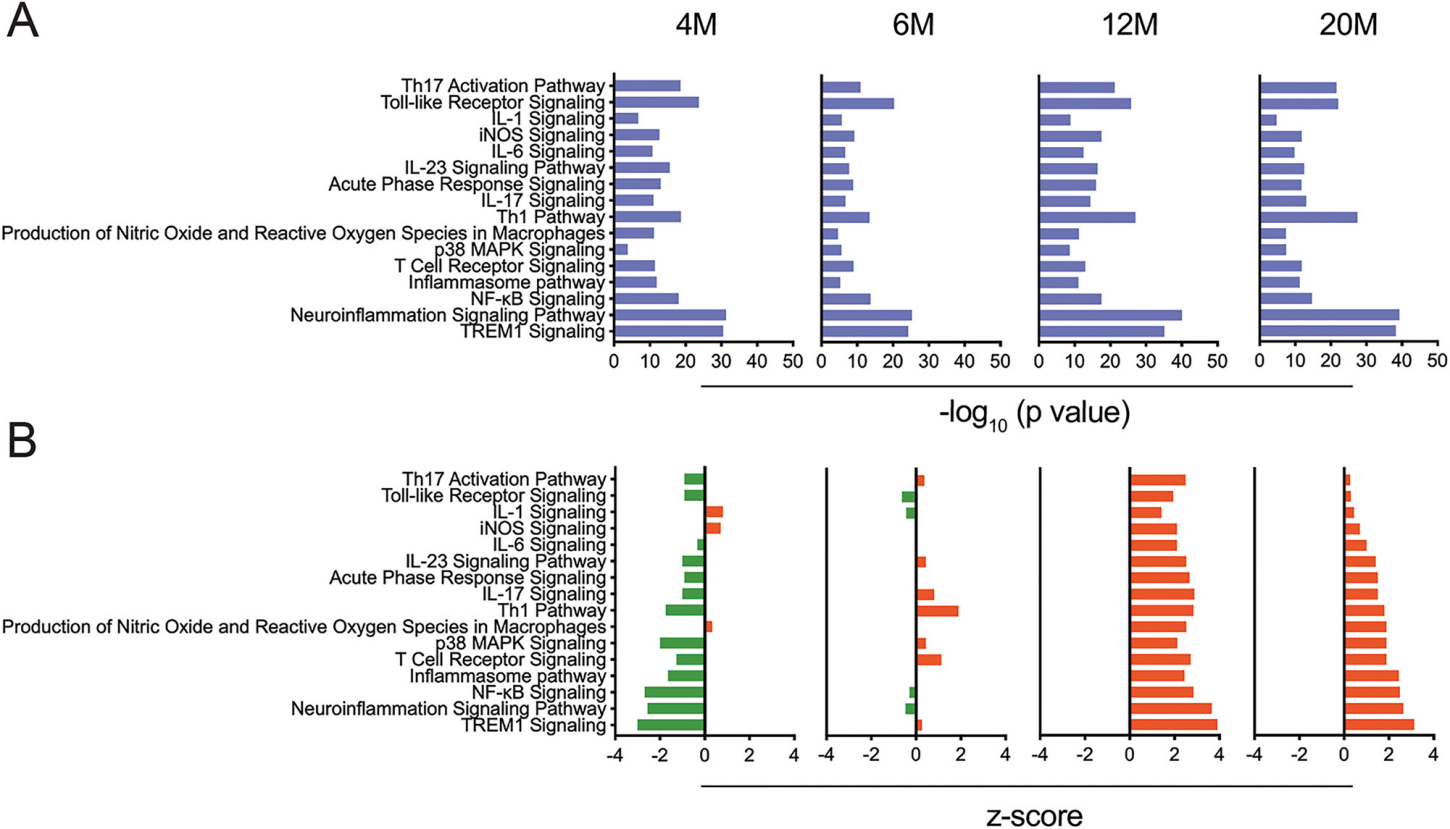
Canonical pathway enrichments of immune-related genes for APP/PS1 mice compared to age-matched non-Tg control mice. Hippocampal tissues were obtained from APPP/PS1 and non-Tg control mice at 4-, 6-, 12- and 20-months of age (4M, 6M, 12M, and 20M). Canonical pathway enrichment analysis was performed using IPA (Qiagen) for APP/PS1 mice of different ages compared to age-matched non-Tg control mice (4M, 6M, 12M, and 20M). (A) Data was represented using −log_10_ (p value) and p values ≤0.05 were considered significant. (B) Data represented as z-scores whereby z-scores ≤−2 or ≥2 were considered significant. Inhibited pathways are represented in green, whereas activated pathways are represented in red.

IPA of immune canonical pathways associated with hippocampal tissues from APP/PS1 mice at different ages compared to those tissues from age-matched non-Tg mice showed significant enrichment of different inflammatory, oxidative stress, and cellular activation pathways that included those associated with TREM1, neuroinflammation, NF-κB, inflammasome, p38 MAPK, IL-1, IL-6, IL-17, IL-23, Th1, Th17 activation, inducible NO synthase (iNOS), production of NO and ROS in macrophages, acute phase response, T cell receptor signaling, and TLR signaling pathways (Figure 5A and Data File 9). Table 2 shows the z and p values of enriched pathways associated with age-related disease progression. A majority of pathways (with the exception of production of NO and ROS in macrophages, IL-1, and iNOS signaling pathways) were inhibited in the 4-month old APP/PS1 mice compared to age-matched non-Tg mice, supporting the absence of cellular activation in 4 month old AD mice with inaugurated amyloid pathology or reactive microglia (Figure 5B and Data File 9). At 6 months, there was a marked shift in the immune phenotype with elevation of pro-inflammatory Th17, Th1 and IL-17 pathways with minimal downregulation of neuroinflammation and NF-κB signaling (Figure 5B and Data File 9). This supports the notion that by 6-months of age, immune cell activation and inflammation had initiated in the hippocampus. The peak inflammatory phenotype was observed at 12-months of age with upregulation of all neuroinflammatory and pro-inflammatory T cell pathways (Figure 5B and Data File 9). Interestingly, at 20 months, while the immune phenotype still remained neuroinflammatory, there was a reduced upregulation of Th17 activation pathway, Toll-like receptor, IL-1, iNOS and IL-6 signaling compared to 12-month mice (Figure 5B and Data File 9). Together, the data indicates that increasing Aβ deposition creates an immunoinflammatory phenotype in the brain with peak upregulation observed at 12-months of age. While the immunophenotype remains pro-inflammatory at 20-months of age, we observe reduced upregulation in Th17 activation pathway, Toll-like receptor, IL-1, iNOS and IL-6 signaling in old mice. This could be because of age-associated increase in Treg function observed in 20-month old mice leading to hippocampal anti-inflammatory responses.

**Table 2:** Progressive AD signaling in APP/PS1 mice of different ages.

Canonical pathways	4-month old		6-month old		12-month old		20-month old	
	z-score	p-Value	z-score	p-Value	z-score	p-Value	z-score	p-Value
Acute phase response signaling	−0.905	1.00 × 10^−13^	0	1.35 × 10^−9^	2.673	1.00 × 10^−16^	1.508	2.00 × 10^−12^
IL-1 signaling	0.816	1.70 × 10^−7^	−0.447	2.34 × 10^−6^	1.414	1.45 × 10^−9^	0.447	1.62 × 10^−5^
IL-17 signaling	−1	1.00 × 10^−11^	0.816	1.91 × 10^−7^	2.887	3.98 × 10^−15^	1.508	7.94 × 10^−14^
IL-23 signaling	−1	2.51 × 10^−16^	0.447	1.86 × 10^−8^	2.53	3.98 × 10^−17^	1.414	3.16 × 10^−13^
IL-6 signaling	−0.333	1.58 × 10^−11^	0	2.51 × 10^−7^	2.111	3.16 × 10^−13^	1	1.78 × 10^−10^
Inflammasome pathway	−1.633	1.26 × 10^−12^	NaN	5.50 × 10^−6^	2.449	1.00 × 10^−11^	2.449	6.31 × 10^−12^
iNOS signaling	0.707	2.00 × 10^−13^	0	6.46 × 10^−10^	2.111	3.16 × 10^−18^	0.707	1.58 × 10^−12^
Neuroinflammation signaling	−2.558	5.01 × 10^−32^	−0.471	5.01 × 10^−26^	3.651	7.94 × 10^−41^	2.6464	5.01 × 10^−40^
NF-κB signaling	−2.673	1.00 × 10^−18^	−0.302	2.00 × 10^−14^	2.84	3.16 × 10^−18^	2.496	2.00 × 10^−15^
p38 MAPK signaling	−2	1.41 × 10^−4^	0.447	3.09 × 10^−6^	2.121	2.34 × 10^−9^	1.89	4.07 × 10^−8^
Production of NO and ROS in macrophages	0.333	6.31 × 10^−12^	0	2.69 × 10^−5^	2.53	6.31 × 10^−12^	1.89	4.68 × 10^−8^
T cell receptor signaling	−1.265	3.98 × 10^−12^	1.134	1.12 × 10^−9^	2.714	1.00 × 10^−13^	1.897	1.58 × 10^−12^
Th1 pathway	−1.732	2.00 × 10^−19^	1.89	3.98 × 10^−14^	2.84	1.00 × 10^−27^	1.807	3.16 × 10^−28^
Th17 activation pathway	−0.905	2.51 × 10^−19^	0.378	1.26 × 10^−11^	2.496	6.31 × 10^−22^	0.277	2.51 × 10^−22^
Toll-like receptor signaling	−0.905	2.00 × 10^−24^	−0.632	5.01 × 10^−21^	1.941	1.58 × 10^−26^	0.302	1.00 × 10^−22^
TREM1 signaling	−3	3.16 × 10^−31^	0.277	6.31 × 10^−25^	3.9	7.94 × 10^−36^	3.13	5.01 × 10^−39^

All comparisons were performed compared against age matched non-Tg controls. The absolute z-score ≥2 and p value ≤0.05 are considered significant. Positive z-score is upregulation, negative z-score is downregulation, z-score of zero is no change in activity. NaN, not a number.

## Discussion

Dysregulation of the adaptive and innate immune system has been implicated in AD progression [49]. In this study, we evaluated age-associated changes in peripheral immune system and immune transcriptomic changes in the hippocampus of APP/PS1 mice, a progressive AD transgenic mouse model. In line with previous reports, we observed age-related increases in amyloidosis and microglia activation as a consequence of disease progression [35, 36, 50–54]. APP/PS1 mice showed significant loss of Treg immunosuppressive function with age in addition to an increased pro-inflammatory hippocampal transcriptomic phenotype. Notably, age-associated improvement in Treg immunosuppressive function observed in 20-month-old mice reduced the hippocampal inflammatory phenotype in old mice. However, improvements in Treg function were not strong enough to either reduce amyloid load or reactive microglia. Our study highlights the therapeutic potential of modulating and restoring Treg function in AD.

Emerging evidence demonstrates that adoptive immune system plays a key role in neuroinflammation and pathobiology of AD [23]. Studies have shown that early transient depletion of Tregs accelerate cognitive impairment and amplification of Tregs through peripheral IL-2 treatment restored cognitive function in APP/PS1 mice [55, 56]. However, changes in Treg function and associated hippocampal immune transcriptomic changes with increasing pathobiology have not been adequately characterized in APP/PS1 mice. Our data show that while Treg numbers are maintained in mice, impairment of Treg immunosuppressive function is a key feature in the development of AD pathobiology. Hippocampal immune transcriptomic evaluation mirrored peripheral Treg dysfunction and showed increasing T cell receptor signaling and Th1 pathways with advancing AD pathogenesis and inflammatory responses peaking in 12-month-old mice. Many inflammatory pathways include TREM1, Th1, NF-κB, IL-17, production of NO and ROS in macrophages, acute phase response, and T cell receptor signaling pathways were also enriched. Additionally, upregulation of pro-inflammatory genes was recorded including *MAPK1*, *TLR2*, *MYD88*, and *NF-κB1.* Each is known to affect AD pathobiology via T cell activation [40, 41, 57], amyloidogenesis [44], neuroinflammation, and neuronal damage [45, 46]. Specifically, enrichment of C3 thioester hydrolysis, a part of the complement cascade, is operative in aged APP/PS1 mice and is known to affect the brain’s homeostatic environment. These findings support prior work demonstrating that complement activation is associated with neurodegeneration and linked to tau pathologies [58]. Additionally, we show increased circulating CD8+ T cells in blood paralleling AD progression. Our data are in agreement with previous reports which demonstrate CD8+ T cell infiltration in the brains of APP/PS1 mice as early as ten months of age and increases thereafter [8, 21]. The immune transcriptomic profile of the hippocampi in APP/PS1 mice compared to age-matched non-Tg control mice showed upregulation of pro-inflammatory cytokines and chemokines with age (Figure 5). This included *CCR4*, *CCR5*, *CXCL8*, *CXCL10*, *CXCR3*, *CRP*, *TLR2*, *TLR4*, *TLR7*, *CCL2*, *IL23A*, *NLRP3*, and *TNF*. Additionally, the data showed enrichment of inflammatory and oxidative stress signaling pathways that include genes encoding for TREM1, NF-κB, p38 MAPK, IL-1, IL-6, IL-17, IL-23, Th1, Th17, iNOS, T cell and TLR signaling pathways.

Interestingly, in 20-month-old mice, there was a marked reduction in the inflammatory profile in the mouse hippocampus compared to 6- and 12-month-old mice. The reduction in hippocampal inflammatory phenotype coincides with age associated improvement in peripheral Treg immunosuppressive function compared to 6- and 12-month-old mice [22]. Studies have shown that Tregs from aged C57BL/6 mice and humans show improved suppression of Teff cell proliferation [22]. Moreover, a significant increase in suppressive activity of Treg cells was found in elderly AD and PD patients [59]. Tregs from aged mice were shown to have a greater redox remodeling-mediated suppression of Teff by reducing the level of extracellular cysteine, producing higher levels of IL-10, and suppressing CD86 expression more than those from young mice [22]. Notably, in our study, the modest improvement in peripheral Treg function was able to significantly reduce the inflammatory phenotype of the hippocampal immune transcriptome with reduced T cell receptor signaling and Th1 pathway. This was despite 20-month-old APP/PS1 mice being at the height of amyloid accumulation and microglial activity. However, the age-associated improvements in Treg immunosuppression function were not robust enough to affect changes in amyloid pathology or reactive microglia. These findings support the notion that promoting Treg immunosuppressive function can serve as a therapeutic modality for AD and reduce neuroinflammation. Indeed, our laboratory and others have shown the therapeutic efficacy of promoting Treg-mediated immunosuppression via pharmaceutical agents and Treg based cell therapies not only in AD, but also in a host of neurological disorders such as PD and ALS [26, 27, 60]. Taken together, the data show that restoring Treg function can lead to therapeutic outcomes for AD [23].

## Conclusions

In summary, the significance of the APP/PS1 model in pre-clinical AD research necessitates understanding the molecular mechanisms and immune responses during disease progression as it relates to that of AD patients [61]. In this study, we unraveled the cellular immune processes and Treg function in AD pathologies. The results demonstrate that Treg dysfunction is a key immunological feature of AD pathology. Age-associated restoration of Treg function in old mice had a marked effect in reducing the inflammatory phenotype of APP/PS1 mice brain, but were not strong enough to reduce amyloid plaque or reactive microglia. Restoration of Treg function via pharmaceutical agents or Treg cell therapeutics can be a potentiation target for disease modifying therapies for AD.

## Supplementary Material

Supplementary Material Details

Supplementary Material Details

Supplementary Material Details

Supplementary Material Details

Supplementary Material Details

Supplementary Material Details

Supplementary Material Details

Supplementary Material Details

Supplementary Material Details

Supplementary Material Details
