# Supplementary material for: Immune senescence in aged APP/PS1 mice
Source: NeuroImmune Pharm Ther. 2023 Aug 14;2(3):317–30. doi: 10.1515/nipt-2023-0015 (PMC10659760; doi:10.1515/nipt-2023-0015)
Supplement: Supplementary file 1 — Supplementary Material Details [file j_nipt-2023-0015_suppl_001.pdf]

*Supplementary Material*

# Immune senescence in aged APP/PS1 mice

Mai M. Abdelmoaty[1*], Pravin Yeapuri[1*], Jatin Machhi[1], Yaman Lu[1], Krista L. Namminga[1], Rana Kadry[2], Eugene Lu[1], Shaurav Bhattarai[1], R. Lee Mosley[1], and Howard E. Gendelman[1#]

[1]Department of Pharmacology and Experimental Neuroscience, College of Medicine, University of Nebraska Medical Center, NE 68198, USA

[2]Department of Cellular and Integrative Physiology, University of Nebraska Medical Center, NE 68198, USA

*Equal contributions

[#]**Corresponding author:** Howard E. Gendelman, Department of Pharmacology and Experimental Neuroscience, University of Nebraska Medical Center, Omaha, NE 6898-5880; phone 402-559-8920; fax 402-559-3744; email hegendel@unmc.edu

## Supplementary Figures

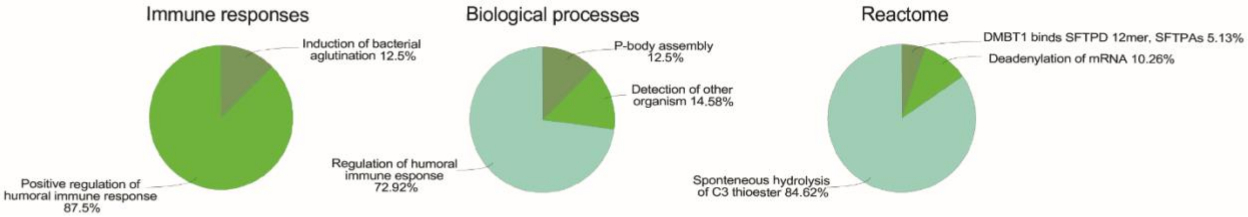


**Supplementary Figure 1. GO annotation of immune-related genes.** Gene enrichment analysis of screened genes from hippocampi of APP/PS1 mice at 6-, 12, and 20-months of age compared to those genes in 4-months old APP/PS1 mice was performed using Cytoscape in conjunction with the plug-in ClueGO. Pie charts represent the distribution of screened genes according to the observed immune responses, biological processes, and Reactome reactions and pathways.

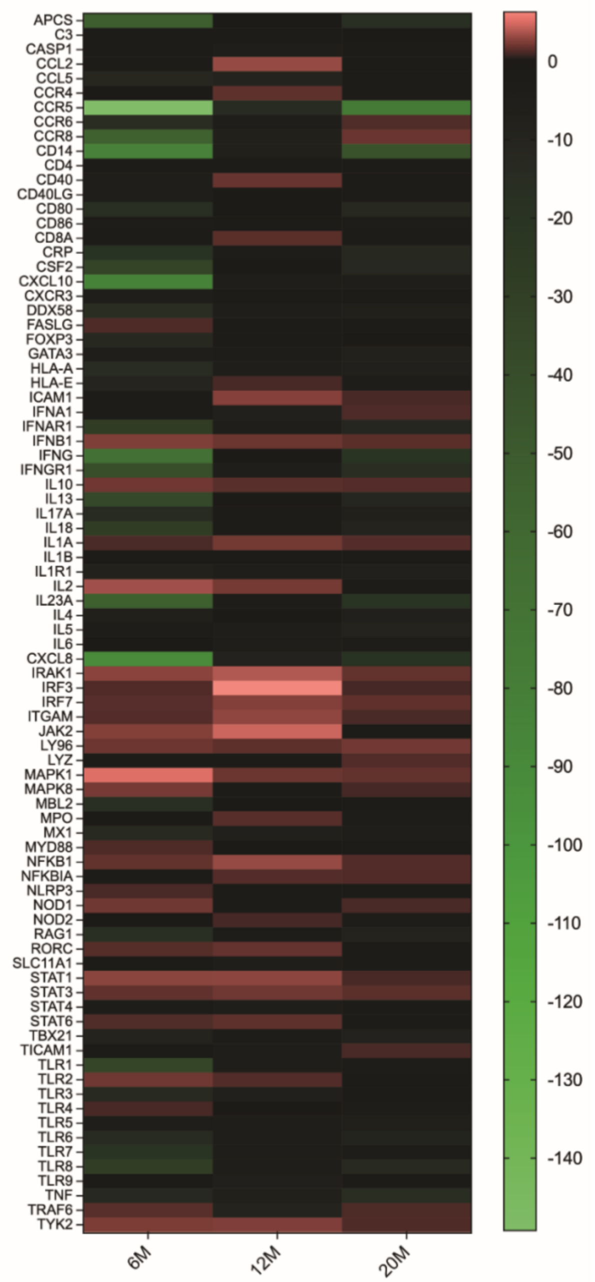


**Supplementary Figure 2. Changes in expression of immune response genes with age for the hippocampus of non-Tg control mice.** Hippocampal tissues were obtained from non-Tg control mice at 4-, 6-, 12- and 20-months of age (4M, 6M, 12M, and 20M, respectively). A heat map representing fold changes in the expression of 84 genes specific for mouse innate and adaptive immune responses in 6M, 12M, and 20M non-Tg mice compared to 4M non-Tg mice (4 mice per group).

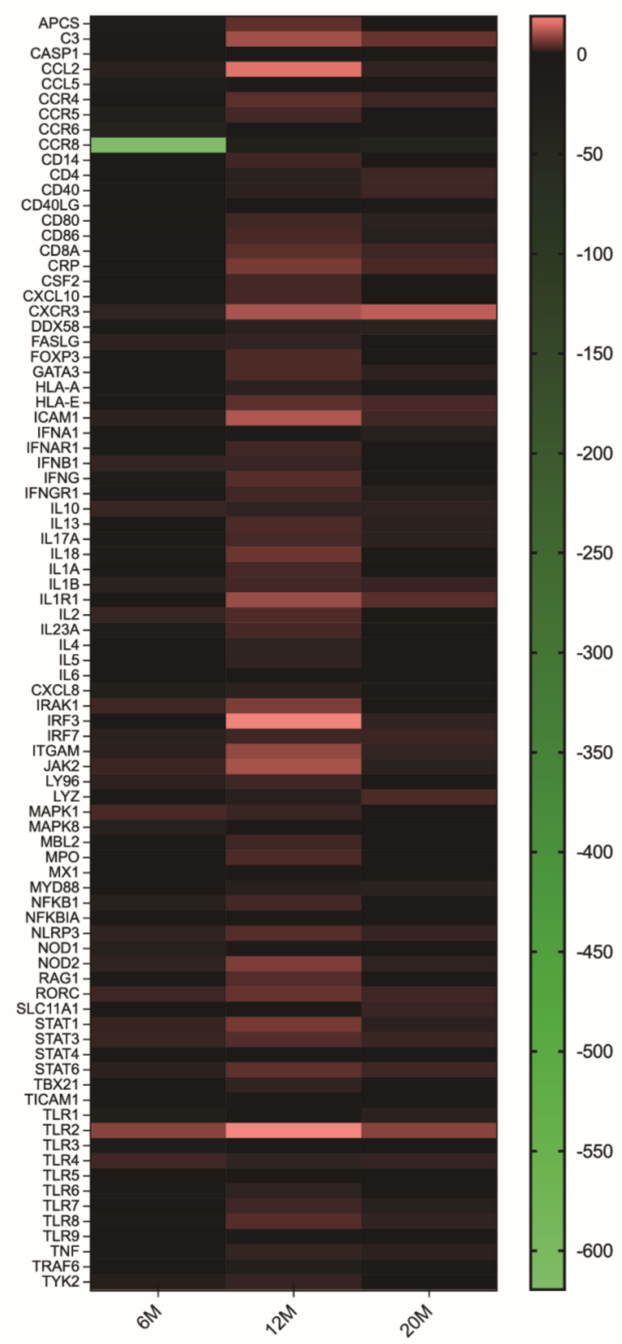


**Supplementary Figure 3. Changes of immune response gene expression for hippocampi from AD mice of different ages.** Hippocampal tissues were obtained from APP/PS1 mice at 4-, 6-, 12- and 20-months of age (4M, 6M, 12M, and 20M, respectively). A heat map representing fold changes in the expression of 84 genes specific for mouse innate and adaptive immune responses in 6M, 12M, and 20M APP/PS1 mice compared to 4M APP/PS1 mice (4 mice per group).

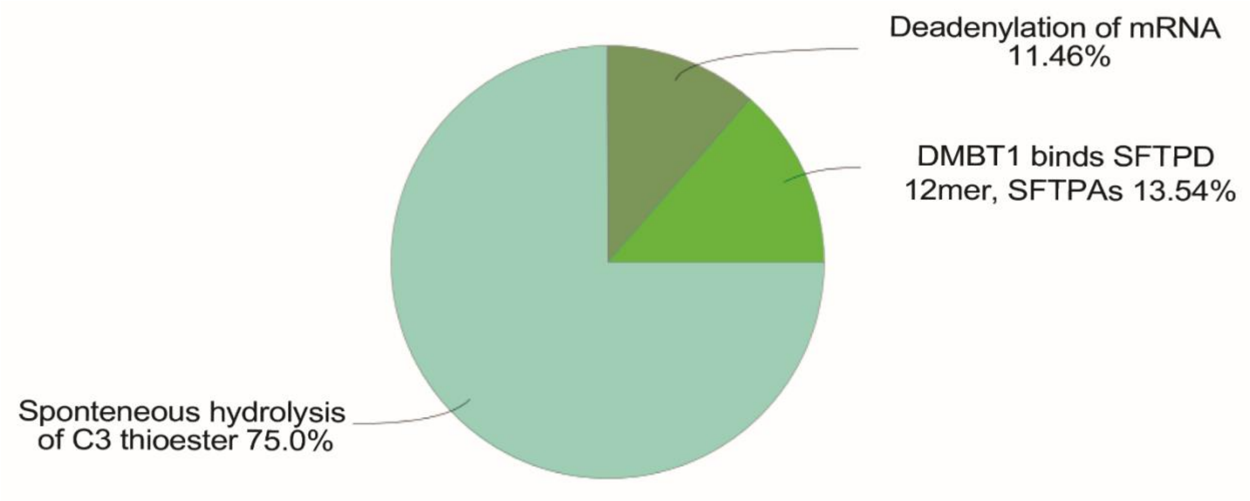


**Supplementary Figure 4. GO annotation of upregulated and downregulated immune-related genes at 12 and 20 months of the disease progression.** Gene enrichment analysis of screened genes from hippocampi of APP/PS1 mice at 12- and 20-months of age compared to 4-months of age was performed using Cytoscape in conjunction with the plug-in ClueGO. Both comparisons showed similar pie charts. Pie charts represent the distribution of screened genes according to the observed immune responses, biological processes, cellular components, molecular functions, and Reactome reactions and pathways.

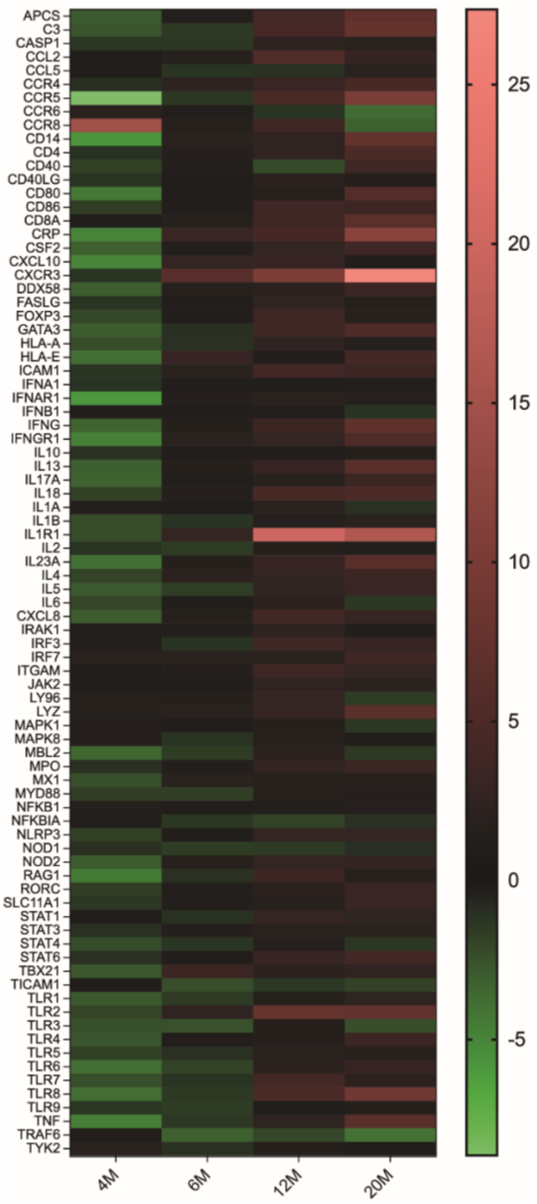


**Supplementary Figure 5. Changes of immune response gene expression in APP/PS1 mice compared to age-matched control mice.** Hippocampal tissues were obtained from APP/PS1 Tg and non-Tg control mice at 4-, 6-, 12- and 20-months of age (4M, 6M, 12M, and 20M, respectively). A heat map representing fold changes in the expression of 84 genes specific for mouse innate and adaptive immune responses in 4M, 6M, 12M, and 20M APP/PS1 mice compared to age-matched non-Tg control mice (4 mice per group).
